# Baseline predictors of remission, pain and fatigue in rheumatoid arthritis: the TITRATE trial

**DOI:** 10.1186/s13075-021-02653-1

**Published:** 2021-11-04

**Authors:** Sook Yan Lee, Fowzia Ibrahim, Brian D. M. Tom, Elena Nikiphorou, Frances M. K. Williams, Heidi Lempp, David L. Scott

**Affiliations:** 1 Centre for Rheumatic Diseases, Department of Inflammation Biology, School of Immunology and Microbial Sciences, Faculty of Life Sciences and Medicine, King’s College London, Cutcombe Road, London, SE5 9RJ UK; 2 MRC Biostatistics Unit, University of Cambridge, East Forvie Building, Forvie Site, Robinson Way, Cambridge Biomedical Campus, Cambridge, CB2 0SR UK; 3 Twin Research & Genetic Epidemiology, School of Life Course Sciences, King’s College London, St Thomas’ Hospital, London, SE1 7EH UK

**Keywords:** Anxiety, Depression, Disease activity score, Fatigue, Intensive management, Obesity, Pain, Rheumatoid arthritis

## Abstract

**Background:**

Clinical trials show intensive treatment to induce remission is effective in patients with highly active rheumatoid arthritis (RA). The TITRATE trial showed that the benefits of intensive treatment also extend to moderately active RA. However, many patients failed to achieve remission or show improvements in pain and fatigue. We investigated whether baseline predictors could identify treatment non-responders.

**Methods:**

The impact of obesity, depression, anxiety and illness perception on RA outcomes, including disease activity, remission, pain and fatigue were determined using a pre-planned secondary analysis of the TITRATE trial data.

**Results:**

Body mass index was associated with disease activity levels and remission: obese patients had a higher overall disease activity and fewer obese patients achieved remission. Intensive management was not associated with increased remission in these patients. Obesity was also associated with increased overall pain and fatigue. Anxiety, depression and health perceptions had no discernible impact on disease activity but were associated with high levels of pain and fatigue. There was a strong association between anxiety and high pain scores; and between depression and high fatigue scores; and health perception was strongly related to both. None of the predictors had an important impact on pain and fatigue reduction in cross-sectional analysis.

**Conclusions:**

Disease activity is higher in obese patients and they have fewer remissions over 12 months. Anxiety, depression and health perceptions were associated with higher pain and fatigue scores. Intensive management strategies need to account for these baseline features as they impact significantly on clinical and psychological outcomes.

**Trial registration:**

ISRCTN 70160382; date registered 16 January 2014

**Supplementary Information:**

The online version contains supplementary material available at 10.1186/s13075-021-02653-1.

## Significance and innovations

Patients with moderately active RA respond heterogeneously to intensive treatment. Disease activity is higher in obese patients: they also have a lower chance of remission. Anxiety, depression and health perception are associated with higher pain and fatigue scores. Intensive treatment strategies need to account for baseline obesity, psychological status and illness perceptions.

## Background

Key treatment goals in rheumatoid arthritis (RA) include minimising disease activity by achieving remission, decreasing disability and improving health-related quality of life [1]. In patients with active RA, particularly those with early disease, intensive treatment using treat-to-target approaches, achieves these goals in many patients [2]. There is more uncertainty whether intensive treatment is equally beneficial in patients with RA with established disease who only have moderate disease activity. The Treatment Intensities and Targets in Rheumatoid Arthritis Therapy (TITRATE) trial assessed whether patients with moderate disease activity would also benefit from intensive treatment [3, 4] in combination with psychosocial support. TITRATE confirmed that intensive treatment does increase remission rates in patients with moderately active established disease.

There is growing recognition that many patients fail to respond to intensive treatments using treat-to-target approaches and understanding the reasons for treatment failure remains an important research objective. There is strong evidence that obesity is a factor influencing response to treatment in RA [5]. In addition, depression and mental health status can influence RA clinical outcomes [6]. Therefore, we evaluated the impact of baseline obesity, mental health status and health perceptions on key clinical outcomes in the TITRATE trial. We focused on three main outcomes: disease activity score, pain, fatigue, and evaluated not only the overall scores, but also important endpoints spanning remission and minimal clinical improvements in pain and fatigue.

## Methods

### Study design

The TITRATE trial protocol and the main trial paper have been published [3, 4]. In brief, the trial design was an open-label, 12-month, pragmatic, randomised, multicentre, two-arm, parallel-group superiority trial. The TITRATE trial was ethically approved by the London—West London & GTAC National Research Ethics Service (NRES) Committee (13/LO/1308). All participants provided written informed consent before participating in the trial.

### Participants

Participants were recruited from 39 specialist rheumatology centres across England; they were all aged over 18 years, met 2010 RA classification criteria [7], had received ≥6 months conventional synthetic disease-modifying anti-rheumatic drugs (csDMARDs), were currently receiving at least one csDMARD and had intermediate disease activity score based on Erythrocyte Sedimentation Rate (DAS28-ESR 3.2-5.1 with at least one swollen joint). Participants were randomised to a standard care (SC) arm or an intensive management (IM) arm, patients in the IM arm received a combination of psychosocial support and drug treatment as per an agreed treatment algorithm. The intervention was delivered by nurses and allied healthcare professionals who completed a 2-day training course on delivering intensive management using motivational interviewing techniques and followed a pre-defined treatment support programme [8–10].

### Assessments

Outcome assessments comprised the disease activity score for 28 joints using the erythrocyte sedimentation rate (DAS28-ESR), pain [11] and fatigue [12] both were measured on 100mm visual analogue scores (VAS). The baseline predictors which spanned lifestyle factors (obesity), mood (depression and anxiety) and health beliefs and illness perceptions were pre-specified in the protocol [3]. Depression and anxiety were measured by Patient Health Questionnaire-9 (PHQ-9) [13] and Generalised Anxiety Disorder-7 (GAD-7) [14], health perception assessed by the Brief Illness Perceptions Questionnaire (BIPQ) [15] and body mass index (BMI, kg/m^2^).

### Statistical methods

All the baseline variables were complete. Remission at 12 months was defined as a 12-month DAS28-ESR < 2.6; ∆ pain at 12 months was calculated as a change in pain score between 12 months and baseline, then it was categorised into two groups (achieving ≥10-unit improvement in pain =1; achieving <10-units worsening in pain =0). At least 10-unit improvement from baseline is classed as a minimal clinical improvement in pain [11]. Similarly, ∆ fatigue at 12 months was calculated as a change in fatigue score between 12 months and baseline, then it was categorized into two groups (achieving ≥10-unit improvement in fatigue =1; achieving <10-units worsening in fatigue =0) [12]. Some patients had missing 12-month outcome data, so this analysis was restricted to patients in whom a 12-month DAS28-ESR, and pain and fatigue scores were available (*n*=299/335 (89%)).

Body mass index (BMI) was calculated as the total body weight (kg) divided by the square of height (m^2^). BMI was categorised according to standard World Health Organization (WHO) definitions: normal = 18.5 to 24.9, overweight = 25.0 to 29.9 and obese ≥ 30.0 kg/m^2^. Of note, there were no patients in this trial who were underweight at <18.5 kg/m^2^.

The depression scale (PHQ-9) was categorised as mild = 0–5, moderate = 6–10, moderately severe = 11–15 and severe depression = 16–20 [13]. Similarly, the anxiety scale (GAD7) was categorised into mild = 0–5, moderate = 6–10, moderately severe = 11–15 and severe anxiety = 16–21 score [14].

Univariable and multivariable logistic regression models were fitted for each binary outcome corresponding to remission, fatigue and pain. Baseline predictors, significant at the 5% level in univariable analyses, were included in multivariable models. In addition, demographic variables (age, gender, ethnicity, disease duration, NHS region) including the trial arm were adjusted for in the multivariable models. Odds ratios (OR) and 95% confidence intervals (CI) were reported for each model.

Furthermore, separate mixed effects models were fitted to each of the three continuous outcomes—DAS28, pain and fatigue. The longitudinal models captured the temporal change at three-time points—baseline, 6 and 12 months. Working correlation matrices were unstructured. Interactions between time and randomised group were assessed in these models. The estimates (*β* coefficients) of magnitude and direction of change in the outcome variables associated with differences in baseline predictor variables, with 95% CI, were reported. All models included random intercepts to account for variation in the outcome variable across individual patients. Follow-up time was included as a factor variable. A random time slope was assessed but found not to be needed as assessed by the likelihood ratio test; thus, the final mixed models included only random intercepts as random effect terms. A mixed effects model dealt with missing outcome data under the missing at random assumption; all patients were included in our analyses, as baseline data were fully observed [16]. Analyses were undertaken using Stata 16 [17].

## Results

### Patients studied

Between August 2014 and July 2017, 459 patients were screened and 335 randomised and treated in the TITRATE trial: 168 patients (140 females) received intensive management and 167 (130 females) had standard care; 134 patients who received intensive management and 124 patients who received standard care completed the trial. 149 patients who received intensive management and 150 who received standard care had all outcome data available and the impact of baseline predictors was assessed in these patients. The mean age was 56 years (SD 12) for both groups, and the mean disease duration was 7 years (SD 7) for intensive management patients and 5 years (SD 5) for patients receiving standard care. Full details of the patients studied are given in Supplementary Table 1.

### Baseline predictors of DAS28, pain and fatigue levels over 12 months

#### Obesity

Longitudinal analyses using mixed-effects models showed patients who were obese at baseline had higher mean DAS28, pain and fatigue scores compared to non-obese patients during the follow-up period. These differences remained significant after adjusting for age, gender, ethnicity, trial arm, disease duration and NHS region (Table 1).

**Table 1:** Influence of baseline BMI on RA disease activity, pain and fatigue over 12 months

		**Crude**		**Adjusted**^a^	
		*β (95%CI)*	*p value*	*β (95%CI)*	*p value*
***Average scores (0, 6 and 12 months)***					
**DAS28-ESR**	*Normal*	Reference		Reference	
	*Overweight*	0.15 (-0.07,0.38)	0.181	0.08 (-0.15,0.31)	0.506
	*Obese*	0.38 (0.14,0.61)	0.002	0.29 (0.05,0.53)	0.017
**Pain**	*Normal*	Reference		Reference	
	*Overweight*	6.00 (0.86,11.13)	0.022	5.17 (0.29,10.05)	0.038
	*Obese*	8.48 (3.36,13.60)	0.001	8.16 (3.12,13.21)	0.002
**Fatigue**	*Normal*	Reference		Reference	
	*Overweight*	3.59 (-2.61,9.79)	0.256	3.36 (-2.05,8.77)	0.224
	*Obese*	7.96 (1.86,14.05)	0.010	5.66 (0.02,11.30)	0.049
***12-month outcomes***					
		**Crude** *Odds ratio (95%CI)*	*p value*	**Adjusted**^a^ *Odds ratio (95%CI)*	*p value*
**Remission DAS28-ESR (<2.6)**	*Normal*	Reference		Reference	
	*Overweight*	0.95 (0.52, 1.70)	0.853	0.97 (0.50, 1.87)	0.925
	*Obese*	0.32 (0.15, 0.67)	0.002	0.33 (0.16, 0.72)	0.005
**Change in Pain (≥10 units)**	*Normal*	Reference		Reference	
	*Overweight*	1.00 (0.58, 1.72)	0.996	1.11 (0.62, 2.00)	0.719
	*Obese*	0.74 (0.42, 1.29)	0.287	0.68 (0.37, 1.24)	0.213
**Change in Fatigue (≥10 units)**	*Normal*	Reference		Reference	
	*Overweight*	0.69 (0.40, 1.18)	0.172	0.76 (0.42, 1.37)	0.362
	*Obese*	0.53 (0.30, 0.94)	0.029	0.53 (0.29, 0.98)	0.043

^a^Adjustment for age, gender, ethnicity, disease duration, NHS region and trial arm; *BMI*: normal *n*=102, overweight *n*=105, obese *n*=92

#### Depression and anxiety

Depression and anxiety were found to be weakly associated with DAS28-ESR in unadjusted models, but the effects became non-significant on adjustment (Tables 2 and 3). Patients who had severe depression at baseline had higher fatigue scores compared to those with no depression after adjusting for baseline factors (Table 2). However, the association of depression with pain seen in the unadjusted model did not persist after adjustment. In contrast, patients with severe anxiety had high levels of pain compared to patients with no anxiety, and this effect persisted after adjusting for baseline factors; though anxiety did not have a substantial impact on fatigue (Table 3) after adjustment.

**Table 2:** Influence of depression on RA disease activity, pain and fatigue over 12 months

		**Crude**		**Adjusted**^a^	
		*β (95%CI)*	*p value*	*β (95%CI)*	*p value*
***Average scores (0, 6 and 12 months)***					
**DAS28-ESR**	*None*	Reference		Reference	
	*Moderate*	0.13 (−0.10, 0.36)	0.278	0.07 (−0.19, 0.33)	0.595
	*Severe*	0.24 (0.02, 0.46)	0.030	0.03 (−0.29, 0.36)	0.838
**Pain**	*None*	Reference		Reference	
	*Moderate*	5.72 (0.66, 10.77)	0.027	0.88 (−4.49, 6.25)	0.749
	*Severe*	11.88 (7.06, 16.71)	<0.001	−0.88 (−7.57, 5.81)	0.796
**Fatigue**	*None*	Reference		Reference	
	*Moderate*	13.12 (7.33, 18.91)	<0.001	10.13 (4.20, 16.07)	0.001
	*Severe*	24.76 (19.56, 29.96)	<0.001	17.07 (9.76, 24.37)	<0.001
***12-month outcomes***					
		**Crude** *Odds ratio (95%CI)*	*p value*	**Adjusted**^a^ *Odds ratio (95%CI)*	*p value*
**Remission DAS28-ESR (<2.6)**	*None*	Reference		Reference	
	*Moderate*	1.28 (0.62, 2.65)	0.500	1.43 (0.64, 3.19)	0.377
	*Severe*	0.78 (0.37, 1.62)	0.498	0.77 (0.35, 1.73)	0.535
**Change in pain (≥10 units)**	*None*	Reference		Reference	
	*Moderate*	0.89 (0.46, 1.72)	0.726	0.66 (0.31, 1.37)	0.264
	*Severe*	1.17 (0.64, 2.13)	0.608	1.19 (0.62, 2.29)	0.597
**Change in fatigue (≥10 units)**	*None*	Reference		Reference	
	*Moderate*	1.61 (0.84, 3.11)	0.152	1.59 (0.76, 3.34)	0.219
	*Severe*	1.25 (0.68, 2.28)	0.469	1.40 (0.72, 2.73)	0.321

^a^Adjustment for age, gender, ethnicity, disease duration, NHS region and trial arm; Depression: none *n*=125; moderate *n*=76; severe *n*=94

**Table 3:** Influence of anxiety on RA disease activity, pain and fatigue over 12 months

		**Crude**		**Adjusted**^a^	
		*β (95%CI)*	*p value*	*β (95%CI)*	*p value*
***Average scores (0, 6 and 12 months)***					
**DAS28-ESR**	*None*	Reference		Reference	
	*Moderate*	0.19 (−0.04, 0.42)	0.097	0.14 (−0.15, 0.42)	0.340
	*Severe*	0.34 (0.11, 0.57)	0.003	0.32 (−0.03, 0.66)	0.071
**Pain**	*None*	Reference		Reference	
	*Moderate*	9.05 (4.01, 14.09)	<0.001	3.44 (−2.76, 9.63)	0.277
	*Severe*	15.03 (9.60, 20.47)	<0.001	9.60 (2.30, 16.90)	0.010
**Fatigue**	*None*	Reference		Reference	
	*Moderate*	15.15 (9.62, 20.68)	<0.001	0.22 (−6.56, 7.00)	0.949
	*Severe*	21.42 (15.08, 27.76)	<0.001	3.12 (−4.39, 10.62)	0.415
***12-month outcomes***					
		**Crude** *Odds ratio (95%CI)*	*p value*	**Adjusted**^a^ *Odds ratio (95%CI)*	*p value*
**Remission DAS28-ESR (<2.6)**	*None*	Reference		Reference	
	*Moderate*	0.74 (0.29, 1.89)	0.528	0.72 (0.25, 2.10)	0.549
	*Severe*	0.79 (0.28, 2.20)	0.647	0.98 (0.33, 2.92)	0.968
**Change in pain (≥10 units)**	*None*	Reference		Reference	
	*Moderate*	0.51 (0.22, 1.16)	0.110	0.46 (0.19, 1.11)	0.083
	*Severe*	2.43 (0.96, 6.16)	0.061	2.88 (0.98, 7.94)	0.046
**Change in fatigue (≥10 units)**	*None*	Reference		Reference	
	*Moderate*	1.11 (0.51, 2.40)	0.790	1.12 (0.48, 2.62)	0.785
	*Severe*	0.99 (0.41, 2.38)	0.985	1.41 (0.54, 3.69)	0.488

^a^Adjustment for age, gender, ethnicity, disease duration, NHS region and trial arm; anxiety: none *n*=183; moderate *n*=65; severe *n*=50

#### Illness beliefs

We found that illness belief, assessed by BIPQ, significantly influenced pain and fatigue scores but not DAS28-ESR (Table 4).

**Table 4:** Influence of illness perception on RA disease activity, pain and fatigue over 12 months

	**Crude**		**Adjusted**^a^	
	*β (95%CI)*	*p value*	*β (95%CI)*	*p value*
***Average scores (0, 6 and 12 months)***				
**DAS28-ESR**	0.01 (−0.001, 0.02)	0.088	0.007 (−0.01, 0.02)	0.306
**Pain**	0.73 (0.50, 0.96)	<0.001	0.62 (0.36, 0.88)	<0.001
**Fatigue**	0.96 (0.68, 1.24)	<0.001	0.51 (0.20, 0.82)	0.001
***12-month outcomes***				
	**Crude** *Odds ratio (95%CI)*	*p value*	**Adjusted**^a^ *Odds ratio (95%CI)*	*p value*
**Remission DAS28-ESR (<2.6)**	1.00 (0.97, 1.03)	0.969	1.01 (0.96, 1.03)	0.808
**Change in pain (≥10 units)**	1.01 (0.98, 1.03)	0.667	1.00 (0.98, 1.02)	0.642
**Change in fatigue (≥10 units)**	1.01 (0.99, 1.04)	0.353	1.02 (0.98, 1.04)	0.387

^a^Adjustment for age, gender, ethnicity, disease duration, NHS region and trial arm

### Predictors of 12-month DAS28-ESR remission

#### Obesity

There was a strong relationship between obesity and remission. The mean BMI was higher in patients not in remission, compared to patients achieving DAS28-ESR remission (29.0 vs 26.0 kg/m^2^, *t* test *p* value = 0.0004), Supplementary Table 2.

**Fig. 1:**
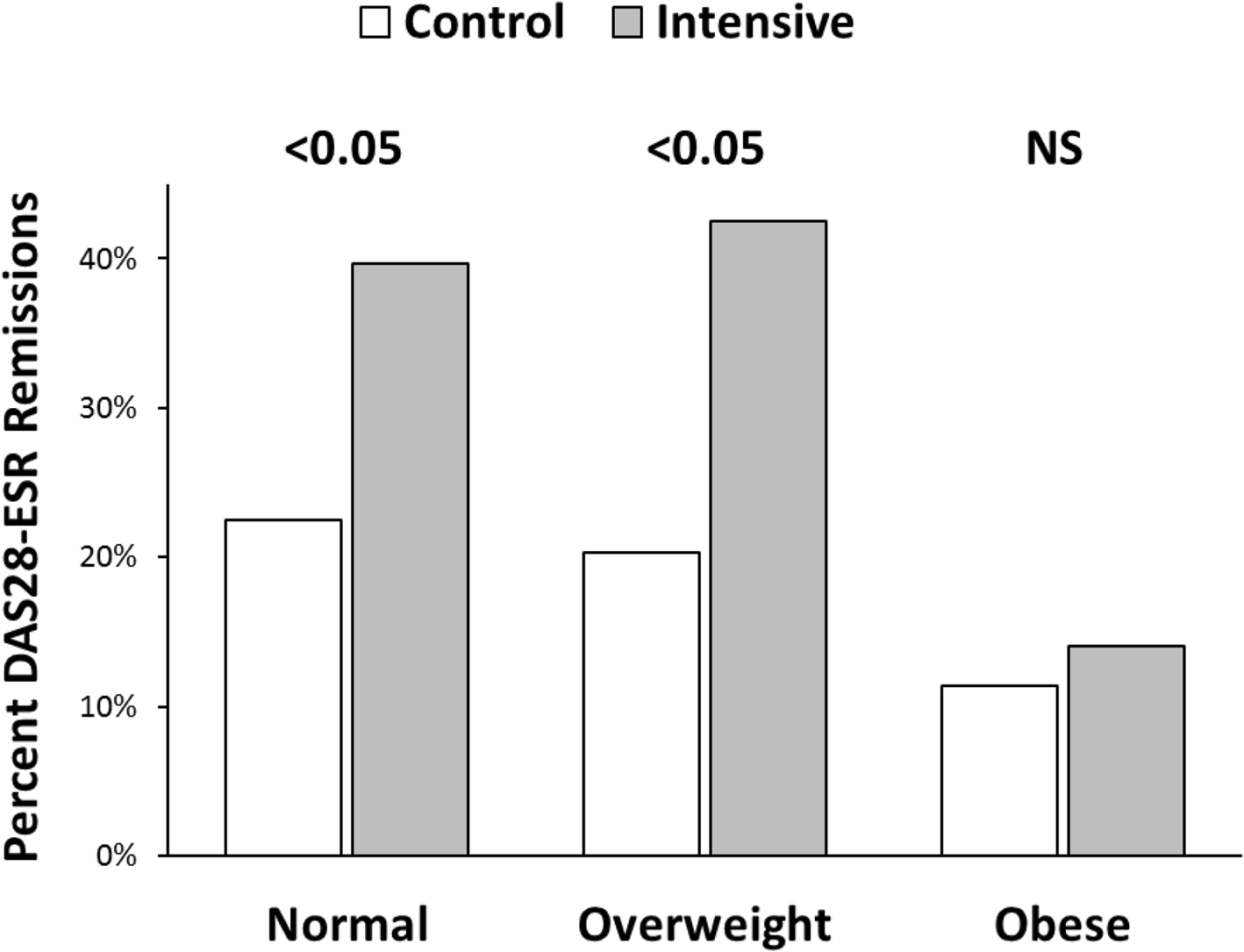
Impact of baseline BMI on 12-month DAS28-ESR remission by obesity status. BMI: normal <25 kg/m^2^, overweight 25–30 kg/m^2^, and obese >35 kg/m^2^. Significance by Fisher’s exact test

Baseline obesity (BMI >30 kg/m^2^) predicted remission at 12 months, and its impact persisted after adjusting for baseline factors (Table 1). Obese patients were less likely to achieve remission compared to those with BMIs <25 kg/m^2^ (adjusted OR 0.34; 95% CI 0.16–0.69, *p* = 0.003). There were more remissions in patients randomised to the intensive management arm, as compared to standard care arm patients who had normal BMIs or were overweight; but there was no significant difference in obese patients (Fig. 1). Further analysis of obese patients showed 5/26 (19%) patients with baseline BMIs of 30–35 kg/m^2^ receiving intensive management achieved remission, as compared with 4/30 (13%) receiving standard care; but only 2/24 (8%) patients with BMIs >35 kg/m^2^ receiving intensive management achieved remission, as compared with 1/14 (7%) receiving standard care.

Detailed analysis of the components of DAS28-ESR at 12 months and changes over 0–12 months (Supplementary Table 5) showed the final ESR was higher in obese patients, compared to those who were of normal weight or were overweight (mean 20.4 vs 13.9; *p*=0.001). The change in the tender joint count, ESR and patient global assessment were lower in obese patients.

An additional analysis evaluated the effects of intensive treatment on changes in DAS28-ESR score in non-obese and obese patients. In non-obese patients, DAS28-ESR scores fell by 0.6 (95% CI 0.3, 0.9) with standard care and by 1.2 (95% CI 0.9, 1.4) with intensive management, and this difference was significant (*p*=0.007); in obese patients, DAS28-ESR scores fell by 0.1 (95% CI −0.2, 0.4) with standard care and by 0.7 (95% CI 0.3, 1.1) with intensive management, and this difference was significant (*p*=0.020). However, final mean DAS28-ESR scores after intensive treatment were lower in non-obese patients (3.2; 95% CI 2.9, 3.5) than in obese patients (3.8; 95% CI 3.4, 4.1), which explains why there were fewer remissions in obese patients. Although the additional analysis shows a fall in DAS28-ESR amongst patients randomised to the intensive management arm, there was no interaction between baseline BMI and the trial arm when examined in the multivariable model (interaction *p* value = 0.482).

#### Depression and anxiety

Mean depression score was higher in patients with active disease as compared to patients in remission (8.4, vs 7.9, *p* = 0.524) (Supplementary Table 2). Slightly more patients with active RA had severe depression (19%), as compared to those with quiescent disease (15%). However, regression analysis showed depression had no important impact on remission outcomes.

Mean anxiety score was higher in patients with active RA (5.4, SD 5.3) as compared to patients with quiescent disease (4.8, SD 5.3) (Supplementary Table 2), but similar proportions of patients had severe anxiety in the remission (7%) and non-remission groups (8%). Regression analysis showed anxiety had no important impact on remission outcomes.

#### Illness beliefs

Illness beliefs had no association on remission rates (Table 4).

### Predictors of 12-month improvements in pain and fatigue

#### Obesity

No evidence of an association between categorised BMI and clinically meaningful changes in pain outcomes was found. Mean baseline BMI was slightly lower in patients whose pain scores improved by ≥10 compared to <10 (27 vs 29, *p* = 0.094) (Supplementary Table 3). Slightly more patients with no reduction in pain levels were obese (34%), as compared to those who did (27%). However, regression analysis showed obesity had no significant impact on improvements in pain (Table 1).

BMI had a stronger relationship to improvements in fatigue. The mean baseline BMI was 27 (SD 6) in patients whose fatigue scores improved by ≥10, as compared with 29 (SD 8) in patients whose fatigue score improved by <10 (Supplementary Table 4). Regression analysis showed obese patients were less likely to reduce fatigue scores as compared to those with BMI <25 kg/m^2^ (Table 1).

#### Depression and anxiety

Baseline depression and anxiety scores were similar for patients regardless of pain outcomes. Regression analyses (Tables 3) showed anxiety scores had a borderline significant (*p*=0.046) impact on pain reduction.

Mean baseline depression and anxiety scores were similar in patients regardless of fatigue outcomes (Supplementary Table 4). Tables 2 and 3 demonstrate that depression and anxiety scores had no significant impact on fatigue.

#### Illness beliefs

Illness beliefs had no association on changes in pain or fatigue (Table 4).

## Discussion

Baseline obesity, depression and anxiety all influence RA clinical and psychological outcome measures, including disease activity, pain and fatigue. However, these symptoms have different impacts. Obese patients had higher overall disease activity measurements and fewer remissions. Obese patients also had higher overall pain and fatigue scores and were less likely to show improvements in fatigue. Anxious patients had higher overall pain levels, while depressed patients had higher overall fatigue scores. However, baseline anxiety and depression were unrelated to improvements in pain and fatigue and had no substantial relationship to RA disease activity level or remission induction.

There is growing recognition that obesity is closely associated with treatment failure and poorer outcomes of not achieving remission when receiving intensive management. Although an association was found between BMI and continuous DAS28-ESR score and remission, the impact of being overweight was minimal; whilst being obese, in particular, having a BMI >35 kg/m^2^ was most clearly associated with a lack of remission. The majority of evidence on the relationship between obesity, disease activity and remission in RA comes from observational studies. A recent study by Schäfer et al. (2020) exemplifies research in this field; they reported the impact of obesity on improvement in DAS28-ESR in 10,593 RA patients in an observational cohort treated with conventional DMARDs and biologics. Obesity reduced the impact of conventional DMARDs and cytokine inhibitors on DAS28-ESR and its components. There are similar findings in many other observational studies [5, 18–28]. In contrast, obesity had no impact on response to the cellular inhibitors rituximab and abatacept [29, 30]. Abuhelwa et al. [31] found a similar impact of obesity on remission, as assessed using the Simple Disease Activity Index (SDAI) and Clinical Disease Activity Index (CDAI), showing that the findings are not confined to assessments using the DAS28-ESR. Systematic reviews have summarised the overall impact of obesity in reducing remission in RA [31–36]. Although there have been fewer analyses of the impact of obesity in clinical trials, those trials which have been analysed report similar findings [37–39] and have the advantage of being longitudinal.

There is uncertainty about why obesity reduces the remission rate with conventional DMARDs and cytokine inhibitors. One possibility is that obese patients might have higher ESR levels, irrespective of their level of systemic inflammation [40]. Another possibility is that clinical assessment and measuring joint counts might be difficult in obese patients [41]. Although we found ESR to be higher in obese patients at 12 months, higher patient global assessment scores were also found, suggesting the impact of obesity on disease activity.

Our detailed analysis of DAS28-ESR change and intensive treatment indicates that obese patients respond to intensive management, but their level of response is reduced as compared to non-obese patients. This is also the case in patients receiving standard care.

The balance of evidence suggests that the difference is either because obese patients have more active disease, or that they are treated insufficiently, though a combination of both is possible. Traditionally, the dose of DMARDs and cytokine inhibitors are not increased in obese patients, and it is possible this method is suboptimal. Dosing by body weight might be a more accurate approach.

The impact of depression and anxiety is different from obesity. There was no evidence either had a major impact on disease activity. However, these two symptoms were associated with higher levels of pain and fatigue throughout treatment, with depression being most associated with fatigue and anxiety with pain. In TITRATE, patient’s pain and fatigue improved with intensive management irrespective of whether they had pre-existing anxiety or depression. However, as the intervention involved both drug and non-drug supportive management, it is possible that the non-drug aspects of management helped reduce psychological symptoms and may have influenced pain and fatigue. This possibility was not evaluated in the trial. Our findings are in keeping with the extensive evidence that anxiety and depression are common in RA compared to the general population and they impact on a range of symptoms, including pain [42, 43]. The interaction between psychological status and disease status in RA is complex [13, 44], and our findings help clarify their impact.

Illness perceptions as assessed by the BIPQ had a comparable impact with both depression and anxiety. All three factors had an influence on the level of pain and fatigue throughout the trial, but were not associated with a significant change in the assessment of these factors at 12 months. Illness perception was also unrelated to DAS28-ESR scores and remission. Similar findings have broadly been reported in recent observational studies of patients with RA who received methotrexate [45] and more intensive treatment [46, 47] for early RA.

The strengths of this study include its sample size, the involvement of many different English centres, and the training of specialist nurses in providing supportive care. The impact of baseline factors on clinical outcomes was key and formed a considered part of the trial design. The study has several limitations. Firstly, PHQ-9, GAD-7 and BIPQ were only assessed at baseline, and there may have been changes during treatment which could have influenced their interactions with pain and fatigue. Secondly, the trial was not powered for the various secondary analyses; consequently, small but potentially important effects of the different predictors may not have been found. Thirdly, we have not assessed the interaction between the different predictors. Although it is likely that assessments of psychological status and health beliefs are inter-related, this trial was too small to evaluate their interaction. A substantially larger study is needed to achieve this goal. Finally, BMI presents a relatively crude assessment of fat mass; the presence of sarcopenia influences the amount of lean body mass and contributes to frailty, which itself is associated with poor clinical/psychological outcomes. Patients diagnosed with RA may have greatly reduced amounts of muscle. More detailed analyses are needed to evaluate this specific issue in detail.

## Conclusions

We conclude that the outcome of RA treatment is influenced by a variety of factors, including obesity, depression, anxiety and illness perceptions. There is a strong case to assess these factors, not only in routine practice but also in future trials of RA management. As obesity reduces remission with conventional DMARDs and cytokine inhibitors given in standard dosage regimens, there is a case for including assessments of baseline obesity in clinical trials of intensive management regimens in RA: Firstly, the extent of obesity may influence the numbers of remission obtained; Secondly, treatment intensity may need to be increased in obese patients.

## Supplementary Information

**Additional file 1: Supplementary Table 1**: Characteristics of the TITRATE trial patients (N=335). **Supplementary Table 2**: Baseline characteristics of patients who achieved remission at 12 months. **Supplementary Table 3**: Baseline characteristics of patients who achieved minimal clinical change in Pain at 12 months. **Supplementary Table 4**: Baseline characteristics of patients who achieved minimal clinical change in Fatigue at 12 months. **Supplementary Table 5**: Impact of Obesity on Components of DAS28-ESR at 12 Months.

## Data Availability

The dataset analysed is not publicly available because data sharing was not part of the original consent and requires institutional approval, but data requests should be submitted to the corresponding author and summary data may be granted following review.

## Abbreviations

BIPQ: Brief Illness Perceptions Questionnaire
BMI: Body Mass Index
CDAI: Clinical Disease Activity Index
CI: Confidence Interval
csDMARD: Conventional Synthetic Disease Modifying Anti-Rheumatic Drug
DAS28: Disease Activity Score For 28 Joints
DAS28-ESR: Disease Activity Score for 28 Joints based on ESR
DMARDs: Disease-Modifying Anti-Rheumatic Drugs
ESR: Erythrocyte Sedimentation Rate
GAD-7: Generalized Anxiety Disorder-7
IM: Intensive Management
NHS: National Health Service
NRES: National Research Ethics Service
OR: Odds Ratio
PHQ-9: Patient Health Questionnaire-9
RA: Rheumatoid Arthritis
SC: Standard Care
SD: Standard Deviation
SDAI: Simple Disease Activity Index
TITRATE: Treatment Intensities and Targets in Rheumatoid Arthritis Therapy Trial
WHO: World Health Organization
