# Supplementary material for: Baseline predictors of remission, pain and fatigue in rheumatoid arthritis: the TITRATE trial
Source: Arthritis Res Ther. 2021 Nov 4;23:278. doi: 10.1186/s13075-021-02653-1 (PMC8567573; doi:10.1186/s13075-021-02653-1)
Supplement: Supplementary file 1 — Additional file 1: Supplementary Table 1: Characteristics of the TITRATE trial patients (N=335). Supplementary Table 2: Baseline characteristics of patients who achieved remission at 12 months. Supplementary Table 3: Baseline characteristics of patients who achieved minimal clinical change in Pain at 12 months. Supplementary Table 4: Baseline characteristics of patients who achieved minimal clinical change in Fatigue at 12 months. Supplementary Table 5: Impact of Obesity on Components of DAS28-ESR at 12 Months. [file 13075_2021_2653_MOESM1_ESM.pdf]

Supplementary Online Material

Supplementary Table 1: Characteristics of the TITRATE trial patients (N=335)

	**Randomised Group**		**Total**
	**IM**	**SC**	
	**n=168**	**n=167**	**n=335**
**Variables**	**Mean (SD)**	**Mean (SD)**	**Mean (SD)**
**Demographics**			
Age at randomisation in years	56.4 (12.2)	56.8 (12.0)	56.6 (12.1)
Disease duration in years	6.6 (7.0)	5.2 (5.5)	5.9 (6.3)
Height (m)	1.65 (0.09)	1.66 (0.09)	1.65 (0.09)
Weight (kg)	77.8 (21.4)	75.9 (15.4)	76.9 (18.7)
Body Mass Index (BMI, kg/m^2)	28.9 (8.5)	27.7 (5.39)	28.3 (7.16)
NHS Region			
London/SE	71 (42.3%)	63 (37.7%)	134 (40.0%)
SW	41 (24.4%)	44 (26.3%)	85 (25.4%)
Midland	19 (11.3%)	21 (12.6%)	40 (11.9%)
NE	37 (22.0%)	39 (23.4%)	76 (22.7%)
Gender, n (%)			
Male	28 (16.7%)	37 (22.2%)	65 (19.4%)
Female	140 (83.3%)	130 (77.8%)	270 (80.6%)
Ethnicity, n (%)			
White	156 (92.8%)	147 (88.0%)	303 (90.4%)
Black	6 (3.6%)	6 (3.6%)	12 (3.6%)
Asian	3 (1.8%)	9 (5.4%)	12 (3.6%)
Mixed/Other	3 (1.8%)	5 (3.0%)	8 (2.4%)
**Clinical**			
Tender Joint counts (28 joints)	7.3 (4.1)	7.5 (4.2)	7.4 (4.1)
Swollen joint counts (28 joints)	4.2 (3.0)	4.1 (2.6)	4.2 (2.8)
Extended Tender Joint counts (68 joints)	12.1 (8.6)	13.2 (8.9)	12.7 (8.8)
Extended Swollen joint counts (66 joints)	5.7 (5.0)	5.4 (3.9)	5.5 (4.5)
Erythrocyte Sedimentation Rate (ESR) (mm/hr)	17.9 (13.9)	15.0 (12.7)	16.5 (13.4)
C-Reactive Protein (CRP) (mg/L)	8.5 (10.7)	7.2 (7.6)	7.8 (9.3)
Assessor global rating (mm)	39.1 (18.2)	41.0 (18.0)	40.0 (18.1)
Patient Global Assessment (mm)	43.3 (19.4)	46.4 (21.4)	44.9 (20.4)
DAS28-ESR	4.4 (0.5)	4.3 (0.5)	4.3 (0.5)
DAS28-CRP	4.5 (0.6)	4.5 (0.6)	4.5 (0.6)
CDAI	19.7 (6.5)	20.4 (6.8)	20.1 (6.6)
SDAI	20.6 (6.3)	21.1 (6.6)	20.8 (6.5)
**Disability**			
Health Assessment Questionnaire (HAQ)	1.2 (0.7)	1.2 (0.7)	1.2 (0.7)
**Psychological**			
Brief Illness Perceptions Questionnaire (BIPQ)	39.9 (8.6)	39.9 (8.7)	39.9 (8.7)
Patient Health Questionnaire-9 (PHQ-9)	8.5 (6.2)	8.4 (6.7)	8.4 (6.5)
Generalised Anxiety Disorder-7 (GAD-7)	5.4 (5.2)	5.3 (5.5)	5.4 (5.3)
**PROMs**			
Pain VAS (mm)	40.4 (22.6)	42.8 (22.5)	41.6 (22.5)

	Randomised Group		Total
	IM	SC	
	n=168	n=167	n=335
**Variables**	**Mean (SD)**	**Mean (SD)**	**Mean (SD)**
Fatigue VAS (mm)	58.9 (25.0)	51.8 (25.3)	55.4 (25.4)

Data are reported as Mean (standard deviation) or number (%).ESR= Erythrocyte Sedimentation Rate; CDAI= Clinical Disease Activity Index; SDAI= Simple Disease Activity Index; VAS= Visual Analogue Scale; DAS28-CRP= Disease Activity Score For 28 Joints based On C-Reactive Protein; DAS28-ESR= Disease Activity Score for 28 Joints based on ESR; IM= intensive management arm; SC=standard care arm; PROMs = patient reported outcome measures

Supplementary Table 2: Baseline characteristics of patients who achieved remission at 12 months

	Yes (N = 76)	No (N = 223)	Total (N = 299)
	**Mean (SD)**	**Mean (SD)**	**Mean (SD)**
Age	56.63 (12.69)	56.68 (11.80)	56.67 (12.01)
Disease duration in years	4.23 (0.52)	4.38 (0.51)	4.34 (0.51)
Gender, n (%)			
Male	21 (27.6%)	36 (16.1%)	57 (19.1%)
Female	55 (72.4%)	187 (83.9%)	242 (80.9%)
Ethnicity			
White	72 (94.7%)	198 (88.8%)	270 (90.3%)
Other ethnic	4 (5.3%)	25 (11.2%)	29 (9.7%)
NHS regions, n (%)			
London/SE	30 (39.5%)	94 (42.2%)	124 (41.5%)
SW	17 (22.4%)	56 (25.1%)	73 (24.4%)
Midland	9 (11.8%)	22 (9.9%)	31 (10.4%)
NE	20 (26.3%)	51 (22.9%)	71 (23.7%)
Trial arm, n (%)			
Standard care	28 (36.8%)	122 (54.7%)	150 (50.2%)
Intensive management	48 (63.2%)	101 (45.3%)	149 (49.8%)
Body Mass Index (BMI, kg/m^2)	25.75 (4.74)	29.15 (7.82)	28.29 (7.31)
WHO BMI categories			
Normal (18.5 to 25.0 kg/m2)	32 (42.1%)	70 (31.4%)	102 (34.1%)
Overweight (25.0 to 30.0 kg/m2)	32 (42.1%)	73 (32.7%)	105 (35.1%)
Obese (>30.0 kg/m2)	12 (15.8%)	80 (35.9%)	92 (30.8%)
Depression score	7.85 (6.70)	8.38 (6.49)	8.25 (6.54)
Depression categories, n (%)			
Mild	33 (44.0%)	92 (41.8%)	125 (42.4%)
Moderate	18 (24.0%)	58 (26.4%)	76 (25.8%)
Moderate sever	13 (17.3%)	28 (12.7%)	41 (13.9%)
Severe	11 (14.7%)	42 (19.1%)	53 (18.0%)
Anxiety	4.80 (5.28)	5.37 (5.28)	5.22 (5.28)
Anxiety categories, n (%)			
Mild	52 (68.4%)	131 (59.0%)	183 (61.4%)
Moderate	13 (17.1%)	52 (23.4%)	65 (21.8%)
Moderate sever	6 (7.9%)	22 (9.9%)	28 (9.4%)
Severe	5 (6.6%)	17 (7.7%)	22 (7.4%)

SW= South West England; NE= North East England; SE= South East England; BMI=body mass index

Supplementary Table 3: Baseline characteristics of patients who achieved minimal clinical change in Pain at 12 months

	≥10 units (N = 138)	<10 units (N = 161)	Total (N = 299)
	**Mean (SD)**	**Mean (SD)**	**Mean (SD)**
Age	57.30 (12.46)	56.12 (11.63)	56.67 (12.01)
Disease duration in years	6.00 (6.84)	5.58 (5.85)	5.77 (6.31)
Gender, n (%)			
Male	26 (18.8%)	31 (19.3%)	57 (19.1%)
Female	112 (81.2%)	130 (80.7%)	242 (80.9%)
Ethnicity			
White	127 (92.0%)	143 (88.8%)	270 (90.3%)
Other ethnic	11 (8.0%)	18 (11.2%)	29 (9.7%)
NHS regions, n (%)			
London/SE	57 (41.3%)	67 (41.6%)	124 (41.5%)
SW	34 (24.6%)	39 (24.2%)	73 (24.4%)
Midland	17 (12.3%)	14 (8.7%)	31 (10.4%)
NE	30 (21.7%)	41 (25.5%)	71 (23.7%)
Trial arm, n (%)			
Standard care	61 (44.2%)	89 (55.3%)	150 (50.2%)
Intensive management	77 (55.8%)	72 (44.7%)	149 (49.8%)
Body Mass Index (BMI, kg/m^2)	27.42 (5.86)	29.03 (8.29)	28.29 (7.31)
WHO BMI categories, n (%)			
Normal (18.5 to 25.0 kg/m2)	50 (36.2%)	52 (32.3%)	102 (34.1%)
Overweight (25.0 to 30.0 kg/m2)	51 (37.0%)	54 (33.5%)	105 (35.1%)
Obese (>30.0 kg/m2)	37 (26.8%)	55 (34.2%)	92 (30.8%)
Depression score	8.07 (6.67)	8.40 (6.44)	8.25 (6.54)
Depression categories, n (%)			
Mild	59 (43.7%)	66 (41.3%)	125 (42.4%)
Moderate	34 (25.2%)	42 (26.3%)	76 (25.8%)
Moderate sever	16 (11.9%)	25 (15.6%)	41 (13.9%)
Severe	26 (19.3%)	27 (16.9%)	53 (18.0%)
Anxiety	5.15 (5.67)	5.29 (4.94)	5.22 (5.28)
Anxiety categories, n (%)			
Mild	92 (66.7%)	91 (56.9%)	183 (61.4%)
Moderate	23 (16.7%)	42 (26.3%)	65 (21.8%)
Moderate sever	8 (5.8%)	20 (12.5%)	28 (9.4%)
Severe	15 (10.9%)	7 (4.4%)	22 (7.4%)

SW= South West England; NE= North East England; SE= South East England; BMI=body mass index

Supplementary Table 4: Baseline characteristics of patients who achieved minimal clinical change in Fatigue at 12 months

	**≥10 units (N = 139)**	**<10 units (N = 160)**	**Total (N = 299)**
	**Mean (SD)**	**Mean (SD)**	**Mean (SD)**
Age	56.02 (11.97)	57.23 (12.07)	56.67 (12.01)
Disease duration in years	5.27 (5.45)	6.21 (6.97)	5.77 (6.31)
Gender, n (%)			
Male	28 (20.1%)	29 (18.1%)	57 (19.1%)
Female	111 (79.9%)	131 (81.9%)	242 (80.9%)
Ethnicity			
White	127 (91.4%)	143 (89.4%)	270 (90.3%)
Other ethnic	12 (8.6%)	17 (10.6%)	29 (9.7%)
NHS regions, n (%)			
London/SE	58 (41.7%)	66 (41.3%)	124 (41.5%)
SW	33 (23.7%)	40 (25.0%)	73 (24.4%)
Midland	14 (10.1%)	17 (10.6%)	31 (10.4%)
NE	34 (24.5%)	37 (23.1%)	71 (23.7%)
Trial arm, n (%)			
Standard care	52 (37.4%)	98 (61.3%)	150 (50.2%)
Intensive management	87 (62.6%)	62 (38.8%)	149 (49.8%)
Body Mass Index (BMI, kg/m^2)	27.23 (6.09)	29.21 (8.12)	28.29 (7.31)
WHO BMI categories, n (%)			
Normal (18.5 to 25.0 kg/m2)	56 (40.3%)	46 (28.8%)	102 (34.1%)
Overweight (25.0 to 30.0 kg/m2)	47 (33.8%)	58 (36.3%)	105 (35.1%)
Obese (>30.0 kg/m2)	36 (25.9%)	56 (35.0%)	92 (30.8%)
Depression score	8.54 (6.50)	7.99 (6.57)	8.25 (6.54)
Depression categories, n (%)			
Mild	55 (40.4%)	70 (44.0%)	125 (42.4%)
Moderate	32 (23.5%)	44 (27.7%)	76 (25.8%)
Moderate sever	24 (17.6%)	17 (10.7%)	41 (13.9%)
Severe	25 (18.4%)	28 (17.6%)	53 (18.0%)
Anxiety	5.16 (5.28)	5.28 (5.30)	5.22 (5.28)
Anxiety categories, n (%)			
Mild	88 (63.3%)	95 (59.7%)	183 (61.4%)
Moderate	28 (20.1%)	37 (23.3%)	65 (21.8%)
Moderate sever	13 (9.4%)	15 (9.4%)	28 (9.4%)
Severe	10 (7.2%)	12 (7.5%)	22 (7.4%)

SW= South West England; NE= North East England; SE= South East England; BMI=body mass index

Supplementary Table 5: Impact of Obesity on Components of DAS28-ESR at 12 Months

	BMI≤30 kg/m² Mean (95% CI)	BMI>30 kg/m² Mean (95% CI)	Significance
Final 12 Month Assessments			
Tender Joint Counts	5.2 (4.4, 6.0)	6.4 (5.3, 7.4)	NS
Swollen Joint Counts	2.8 (2.3, 3.3)	2.4 (1.9, 3.0)	NS
ESR	13.9 (12.2, 15.7)	20.4 (16.3, 24.5)	p=0.006
Patient Global	33.4 (29.9, 37.0)	38.9 (33.8, 44.1)	NS
Change in Assessments 0-12 Months			
Tender Joint Counts	2.5 (1.7, 3.3)	0.6 (-0.6, 1.8)	p=0.013
Swollen Joint Counts	1.5 (1.0, 2.0)	1.3 (0.7, 1.9)	NS
ESR	1.5 (0.0, 2.9)	-1.8 (-4.3, 0.6)	p=0.024
Patient Global	12.4 (8.3, 16.6)	4.2 (-1.5, 9.9)	p=0.023

NS= non- Signiant; p-value from t-test (comparing two means); ESR= Erythrocyte Sedimentation Rate
